# The Reorganization of Rice Rhizosphere Microbial Communities Driven by Nitrogen Utilization Efficiency and the Regulatory Mechanism of Soil Nitrogen Cycling

**DOI:** 10.3390/microorganisms13092215

**Published:** 2025-09-22

**Authors:** Zhuang Xiong, Qiang Li, Rongtao Fu, Jian Wang, Daihua Lu, Cheng Chen

**Affiliations:** 1 Institute of Plant Protection, Sichuan Academy of Agricultural Sciences, Chengdu 610066, China; xiongzhuang2000@126.com (Z.X.); furongtao@126.com (R.F.); 2020101018@stu.sicau.edu.cn (J.W.); ludaihua@scsaas.cn (D.L.); 2 Key Laboratory of Coarse Cereal Processing, Ministry of Agriculture and Rural Affairs, Sichuan Engineering & Technology Research Center of Coarse Cereal Industrialization, School of Food and Biological Engineering, Chengdu University, Chengdu 610106, China; liqiang02@cdu.edu.cn

**Keywords:** nitrogen use efficiency, nitrogen-fixing bacteria, microbial community, soil

## Abstract

Nitrogen use efficiency (NUE) in rice cultivation is a key determinant of sustainable agriculture, yet the interaction between NUE and the dynamics of rhizosphere soil microbial communities remain poorly understood. In this study, the changes in rhizosphere soil microbial community composition and function due to NUE were analyzed in six rice genotypes across six treatments. Through 16S rRNA/ITS amplicon sequencing, it was found that rice with different NUEs reshaped the rhizosphere soil microbial community structure, but did not significantly alter the α-diversity of the bacterial community. There was a notable difference in the average abundance of the fungus *Arnium* in the rhizosphere soil of high-NUE rice compared to low-NUE rice, with a 222.2% increase in the former. Correlation analysis indicated that in high-NUE rice, soil nitrate and nitrite contents drove changes in the fungal community, while in low-NUE rice, soil water-soluble nitrogen and total potassium contents were the key influencing factors for changes in the fungal and nitrogen-fixing bacterial communities, respectively. The findings of this study demonstrate a link between NUE-induced changes in the rhizosphere soil microbiome and nitrogen cycling in rice, providing a basis for targeted nitrogen fertilizer management approaches guided by microbial control.

## 1. Introduction

Nitrogen plays a crucial role in plant growth, and its use efficiency is directly related to agricultural productivity and ecological sustainability [1]. One of the core challenges facing global food security is reducing the negative impact of intensified agriculture on the environment while meeting the needs of a growing population [2]. Nitrogen deficiency is one of the most important causes of global crop yield limitations, and the widespread use of nitrogen fertilizer has been instrumental in enhancing global crop yields [3,4,5]. However, only a small part of the nitrogen fertilizer entering the farmland is effectively absorbed and used by crops, while the remaining part is lost through ammonia volatilization, nitrate leaching, and N_2_O emission caused by nitrification-denitrification [6,7,8,9,10,11]. This large loss of nitrogen not only wastes economic resources but also poses a serious threat to ecosystem health. Therefore, effective nitrogen use in crop production helps improve crop yield and quality, environmental safety, and economic benefits [12,13].

Rice (*Oryza sativa* L.) is a crucial food crop worldwide, and the vast majority of the global population depends on rice as the main source of energy [14]. With over half of the world’s population relying on it as a staple food, the efficiency of nitrogen fertilizer use is generally less than 40%, which means that more than 60% of the applied nitrogen will enter the natural environment, seriously affecting the health of the ecosystem [15,16]. Recent studies have found that there are significant differences in NUE among rice genotypes. These differences are not only related to the genetic characteristics of the host, such as root configuration and expression of nitrogen transporters, but are also deeply regulated by the composition and activity of the rhizosphere microbial community [17,18,19]. In the study by Sun et al. [20], *Proteobacteria*, *Bacteroidota*, *Nitrospirota*, and *Zixibacteria* were identified as the dominant communities in rice with high nitrogen uptake levels. Farooq et al. [21] reported that, based on the results of microbial abundance and structural analysis, the structural composition of ammonia-oxidizing archaea remained unchanged in response to variations in soil and nitrogen fertilizer application. Chen et al. [22] found that under low nitrogen stress, the microbial community diversity of the root-specific promoter transgenic rice root systems underwent significant changes. The synthetic microbial community, composed of six nitrogen-fixing strains, significantly enhanced the nitrogen-fixing ability of the rice root systems. Liu et al. [23] observed a significant increase in the relative abundance of *Methylosinus* in the root systems of rice plants treated with nitrogen fertilizer, and the nitrogen-fixing bacterial community clearly aggregated. The rhizosphere, as a key interface for plant-microbial interactions, significantly influences nitrogen cycling pathways through processes such as nitrogen fixation, organic nitrogen mineralization, and nitrification inhibition [19,24,25]. Gaspareto et al. found that inoculation with *A. brasilense* and *B. subtilis* promoted wheat root development, likely due to their strong biological nitrogen-fixing capabilities, ultimately enhancing nitrogen use efficiency [26]. Liu et al. found that endophytic bacteria in rice promote the reduction of nitrate to ammonium, thereby enhancing nitrogen use efficiency. They also alter the structure of soil microbial communities through synergistic interactions, indirectly influencing nitrogen cycling [27]. However, the way in which the soil environment and microbial community assembly synergistically drive differences in rice NUE, as well as the specific contributions of bacteria, fungi, and nitrogen-fixing bacteria in the microbial community, remains a core issue that has not been systematically elucidated. Currently, despite the extensive research conducted on the physiological mechanisms of rice varieties with varying NUE levels [28,29,30,31], there is a need for additional studies to examine the impact of these rice varieties on soil ecosystems, particularly on the intricate structure, diversity, and essential ecological functions of soil microbial communities.

This study utilized 16S rRNA high-throughput sequencing to examine variations in the diversity and richness of root-associated microbial communities and functional changes among rice varieties with varying NUE levels. At the same time, alterations in soil physicochemical properties were examined to pinpoint the main factors that impact the development of root-associated microbial communities in rice. The study results will provide microbiome targets for breeding nitrogen-efficient rice varieties and lay the theoretical foundation for precision nitrogen fertilizer management strategies based on rhizosphere microbial regulation.

## 2. Materials and Methods

### 2.1. Experimental Material and Design

The experiment was conducted at the Sichuan Academy of Agricultural Sciences in 2025 (104°11′ E, 30°62′ N, China). The rice seeds of the six different varieties used were all from the Plant Protection Institute of Sichuan Academy of Agricultural Sciences. The rice varieties used were selected from preliminary experiments for their excellent growth potential and disease resistance (JXY91, CLYXD, HXY872, HY2986, HY7986, and HY685). The experiment was set up with two experimental groups: a normal nitrogen application group and a nitrogen-free treatment group. Each experimental group had a total of six treatments (six different rice varieties, named C101, C102, C103, C104, C105, and C106, respectively). Each treatment was replicated three times. A total of 36 plots were set up in the same field parcel. With a length of 7 m and a width of 1 m, each plot had an area of 7 m^2^. An interval of 50 cm was maintained between replicates to facilitate observation and agricultural activities in the field. Each variety of rice was arranged randomly. Seedlings were raised using conventional methods in paddy fields. Manual transplanting at the 3–4-leaf stage with a row spacing of 25 cm and a plant spacing of 15 cm was used. Double transplanting was performed. In each plot, there were four rows planted with 46 holes in each row, resulting in 368 seedlings in each plot. During the experiment, prevention and control measures were implemented in a timely manner according to the occurrence of diseases, insects, and weeds.

### 2.2. Determination of the Agronomic Use Efficiency of Nitrogen Fertilizer

The fertilization amount per hectare in the normal nitrogen fertilization group was as follows: 180 kg N + 45 kg P_2_O_5_ + 135 kg K_2_O. Urea is selected as the nitrogen fertilizer and should be applied in several portions in a ratio of 4:3:3 for the base fertilizer, tillering fertilizer, and panicle fertilizer. Superphosphate was chosen as the phosphate fertilizer, with potassium chloride chosen as the potassium fertilizer. Both are applied as base fertilizers. The nitrogen-free treatment group did not receive nitrogen fertilizer, but the same phosphorus and potassium fertilizers were applied in the base fertilizer as the normal nitrogen application group. After the rice is mature, harvest it in a timely manner, dry it in the sun, and remove any impurities. The actual yield of rice in each plot covering a total area of 7 square meters should be measured. Calculate the NUE by referring to the method of He et al. [32].

NUE = (N uptake in N applied area − N uptake in no N applied area)/N applied

### 2.3. Measurement of Soil Physicochemical Properties

Use shovels to collect soil from the 0–20 cm soil layer of the normal nitrogen application group. For each plot, use the 5-point sampling method to collect three times. Mix the collected soil evenly and place it in a sealed bag, then take it back to the laboratory immediately. Remove plant roots, gravel debris, and other impurities from the soil sample, sieve it through a 2 mm mesh, and store it at 4 °C in a refrigerator. The samples will be used to determine the soil pH, organic carbon (OC), total nitrogen (TN), total phosphorus (TP), total potassium (TK), hydrolyzable nitrogen (HN), available phosphorus (AP), available potassium (AK), ammonium nitrogen (NH_4_^+^-N), nitrate nitrogen (NO_3_^−^-N), and nitrite nitrogen (NO_2_^−^-N) content.

Soil pH was determined with a pH meter (FE28, METTLER-TOLEDO, Columbus, OH, USA). The potassium dichromate oxidation-external heating method was used to determine the soil OC content [33]. The soil TN content was analyzed with a fully automatic Kjeldahl analyzer (KN-520, Alva, CN, USA). The soil TP content was measured by the NaOH alkali fusion and molybdenum antimony spectrophotometric method [34]. The soil TK content was measured by the NaOH alkali fusion and flame photometer method [35]. The alkaline hydrolysis diffusion method was employed to determine the soil HN content [36]. The soil AP content was extracted using an ammonium fluoride-hydrochloric acid solution and analyzed using the molybdenum-antimony resistance colorimetric method [37]. The soil AK content was measured by using an ammonium acetate extraction-flame photometer [37]. The soil NH4^+^-N content was extracted with a potassium chloride solution and determined via the indophenol blue colorimetric method. The soil NO_3_^−^-N content was measured using the dual-wavelength colorimetric method after extraction with a potassium chloride solution. The soil NO_2_^−^-N content was measured using the N-(1-naphthyl)-ethylenediamine colorimetric method [38].

### 2.4. DNA Extraction and Sequencing of Rice Rhizosphere Soil Microorganisms

Excavate the soil around the entire root system of the rice plants in the normal nitrogen application group. Shake off excess soil, collect the soil adhering to the roots, and sieve it using a 1 mm mesh screen to remove impurities. Finally, place the sample in a sterile centrifuge tube, freeze it rapidly with liquid nitrogen, and store it at −80 °C. Subsequently, perform DNA extraction and 16S rRNA and ITS sequencing according to standard procedures. Total genomic DNA was obtained from soil samples by using a DNA extraction kit. After extracting the DNA, it was loaded onto a 1% (*w*/*v*) agarose gel to determine its quality. The quantitative PCR procedure was conducted following the method detailed by Xiong et al. [39]. The 16S V3 and 4 region of these samples was amplified with primers 341F (CCTAYGGGRBGCASCAG) and 806R (GGACTACNNGGGTATCTAAT) [40]. The ITS1-5F region of these samples was amplified with primers 1737F (GGAAGTAAAAGTCGTAACAAGG) and 2043R (GCTGCGTCTTCATCGATGC) [41]. In addition, the nifH gene fragment was amplified using primers nifHF (AAAGGYGGWATCGGYAARTCCACCAC) and nifHR (TTGTTSGCSGCRTACATSGCCATCAT) [42]. The construction and sequencing of the amplicons were completed by Beijing Novogene Technology Co., Ltd.(Beijing, China). For detailed methods, please refer to the Supplementary Files.

### 2.5. Statistical Analysis

The experimental data were analyzed using Microsoft Excel. IBM SPSS Statistics 26.0 software was utilized to analyze significant differences through Duncan’s multivariate and independent samples *t*-tests. *p* < 0.05 shows that there is a statistically significant difference between two groups.

## 3. Results

### 3.1. Determination of the Agronomic Use Efficiency of Nitrogen Fertilizer in Rice

Table 1 lists the NUE of different rice varieties. Among all rice varieties, the NUE of the C102 group was the highest at 26.80 ± 0.99 kg/kg. The NUE of the C106 group was the lowest at 11.48 ± 2.37 kg/kg. According to the response of rice to NUE, the rice varieties were divided into high-NUE and low-NUE groups. Among them, the NUE of groups C101, C102, and C103 was significantly greater than that of groups C104, C105, and C106 (*p* < 0.05). Based on these results, groups C101, C102, and C103 were classified as high-NUE groups, while groups C104, C105, and C106 were classified as low-NUE groups.

### 3.2. Changes in Soil Physicochemical Properties

The physicochemical properties of the soil in the different treatment groups are listed in Table 2. Among all treatment groups, groups C101, C102, and C104 had significantly higher pH values compared to the other groups (*p* < 0.05), with group C106 showing the lowest pH value of 5.76 ± 0.03. Although the soil OC contents of groups C101 and C103 were significantly greater than those of groups C102 and C104 (*p* < 0.05), the difference in soil OC content between the high-NUE groups and the low-NUE groups as a whole was not significant. Notably, the soil TN content varied significantly among the treatment groups. Group C103 had a significantly higher soil TN content compared to the other groups, whereas groups C101 and C102 had significantly lower soil TN content than groups C104, C105, and C106 (*p* < 0.05). Group C101 had the highest soil TP content, while group C102 had the lowest content, significantly lower than all other groups (*p* < 0.05). Group C104 had a significantly higher soil TK content compared to the other groups, whereas group C106 had a significantly lower content than the remaining groups (*p* < 0.05). Group C103 showed significantly greater soil HN content compared to the other groups, similar to the results for soil TN content (*p* < 0.05). Group C104 had a significantly higher soil AP content compared to the other groups, whereas group C103 had a significantly lower content than the other groups (*p* < 0.05). The soil AK content in group C103 was significantly higher compared to the other groups, whereas the content in group C101 was significantly lower than the other groups (*p* < 0.05). In terms of soil NH_4_^+^-N content, group C104 had significantly lower soil NH_4_^+^-N content compared to the other groups (*p* < 0.05). There were significant differences among the groups in terms of soil NO_3_^−^-N content. The NO_3_^−^-N content in group C101 was significantly higher than in the other groups, whereas the NO_3_^−^-N content in group C102 was significantly lower than in the other groups (*p* < 0.05). In terms of soil NO_2_^−^-N content, the differences among the groups were relatively small, with only the content of group C101 being significantly greater than that of the other groups (*p* < 0.05).

### 3.3. Changes in the α-Diversity of Rice Rhizosphere Soil Microbes

#### 3.3.1. High-Throughput Sequencing Data Analysis and Microbial Community Composition

First, split the databased on barcodes to obtain the raw data for each sample, removing barcodes and primers. Subsequently, use FLASH software (V1.2.11) to assemble the R1 and R2 sequence data. Perform quality control on the assembled tags to obtain Clean Tags. After removing chimeras, low-quality reads, and short sequences, the average clean reads per bacterial sample for downstream analysis was 91,571. The average clean reads for each fungal sample used in downstream analysis was 95,925. Each nitrogen-fixing bacterial sample yielded an average of 173,440 clean reads for downstream analysis. These clean reads were then clustered into ASVs at a similarity threshold of 97%. There were a total of 68 phyla, 162 classes, 337 orders, 467 families, and 826 genera identified in all the rice rhizosphere soil bacterial samples. At the genus level, the most common species were *Anaeromyxobacter*, *Candidatus Nitrosotalea*, and *ADurb.Bin063-1* (Figure S1). A total of 14 phyla, 42 classes, 92 orders, 181 families, and 270 genera were identified among all the rice rhizosphere soil fungal samples. At the genus level, *Pseudeurotium* had the highest abundance, followed by *Arnium* and *Fungi gen Incertae sedis* (Figure S2). A total of 27 phyla, 66 classes, 120 orders, 208 families, and 375 genera were identified for nitrogen-fixing bacteria. At the genus level, *Bradyrhizobium*, *Anaeromyxobacter*, and *Sideroxyarcus* accounted for a high proportion (Figure S3).

#### 3.3.2. Changes in the α-Diversity of Soil Microbes in the Rice Rhizosphere

**Figure 1:**
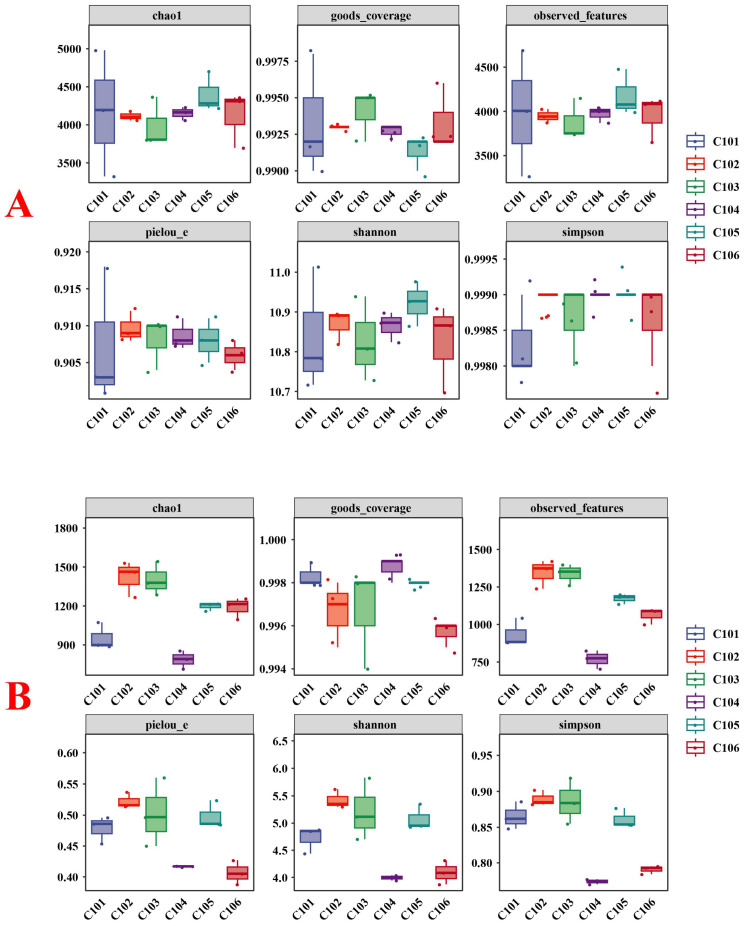
Box plots of α-diversity indices of rhizosphere soil bacterial (**A**) and fungal (**B**) in different treatment groups.

Figure 1 shows the indices used to assess the diversity and richness of rice rhizosphere soils. For rice rhizosphere soil bacteria, no significant changes were detected in any index among all treatment groups, indicating that rice varieties with different NUE did not significantly change the diversity or richness of rhizosphere soil bacteria. However, there were significant changes in the fungal communities of the rhizosphere soil among the rice varieties. The Shannon index and Pielou_e index of groups C102 and C103 were significantly higher than those of groups C104 and C106 (*p* < 0.05), indicating that the fungal diversity of groups C102 and C103 was higher than that of groups C104 and C106. Additionally, the Simpson index of groups C101 and C102 was significantly higher than that of groups C104 and C106 (*p* < 0.05). The Chao1 index and observed features index of groups C102 and C103 were significantly higher than those of group C104 (*p* < 0.05). Overall, the fungal diversity in the rhizosphere soil of rice with high-NUE was higher than that of rice with low-NUE. The changes in nitrogen-fixing bacteria in the rice rhizosphere soil in each treatment group are shown in Figure S4. As shown in the figure, there were no significant differences in any indicators of nitrogen-fixing bacteria in the rhizosphere of rice among the groups. This indicates that rice plants with different NUE did not significantly change the diversity or richness of nitrogen-fixing bacteria in their rhizosphere soil.

#### 3.3.3. Composition and Abundance of Microbes in the Rice Rhizosphere

Among all the samples, the composition and abundance changes in the top ten most abundant bacteria, fungi, and nitrogen-fixing bacteria were compared (Figures S5–S7). *Proteobacteria* is the phylum with the highest abundance, accounting for 22.40% of the total. In group C102, the abundance of *Proteobacteria* was significantly higher compared to groups C103 and C105 (*p* < 0.05). *Acidobacteriota*, the second most abundant phylum, makes up 12.89% of the total. Among them, the abundance of *Acidobacteriota* in groups C103 and C105 was significantly higher than that in group C102 (*p* < 0.05). *Desulfobacterota* is the third most abundant phylum, accounting for 12.12% of the total. Except for group C104, the abundance of *Desulfobacterota* in group C106 was significantly higher than that in the other groups (*p* < 0.05). At the genus level, *Anaeromyxobacter* had the highest abundance, accounting for 3.34% of the total. Except for group C104, the abundance of *Anaeromyxobacter* in group C102 was significantly higher than that in the other groups (*p* < 0.05). Secondly, *Candidatus Nitrosotalea* was the second most abundant genus, accounting for 1.80% of the total. *ADurb.Bin063-1* is the third most abundant genus, accounting for 1.39% of the total.

*Ascomycota* was the most abundant phylum among fungi, representing 31.31% of the total. The abundance of *Ascomycota* in group C101 significantly exceeded that in the other groups (*p* < 0.05). The *Rozellomycota* phylum was the second most abundant, making up 4.29% of the total. The abundance of *Rozellomycota* in groups C102 and C104 was significantly greater than that in the other groups (*p* < 0.05). The third most abundant phylum was *Fungi phy Incertae sedis*, accounting for 3.17% of the total. The abundance of *Fungi phy Incertae sedis* in groups C101 and C105 was notably higher than in the other groups (*p* < 0.05). At the genus level, *Pseudeurotium* had the highest abundance, accounting for 16.25%. Among them, the abundance of *Pseudeurotium* in groups C101 and C106 was significantly greater than that in the other groups (*p* < 0.05). The second most abundant genus was *Arnium*, which made up 6.33% of the total. With the exception of group C101, the abundance of *Arnium* was significantly greater in group C102 than in the other groups (*p* < 0.05). The third most abundant genus was the *Fungi gen Incertae sedis*, accounting for 3.17% of the total. The abundance of the *Fungi gen Incertae sedis* in both groups C101 and C105 was significantly greater than in the other groups (*p* < 0.05).

Among the nitrogen-fixing bacteria, *Pseudomonadota* was the most abundant phylum, accounting for 45% of the bacteria. The abundance of *Pseudomonadota* in group C103 was significantly lower than that in the other groups (*p* < 0.05). The second most abundant phylum was *Unclassified*, accounting for 19% of the total. The third most abundant phylum was *Thermosulfobacteriota*, accounting for 14%. The abundance of *Thermosulfobacteriota* was significantly higher in group C103 compared to the other groups (*p* < 0.05). At the genus level, *Bradyrhizobium* had the highest abundance and accounted for 21% of the bacteria. *Bradyrhizobium* abundance was significantly higher in group C102 compared to the other groups, except for group C106 (*p* < 0.05). The genus *Unclassified* was the second most abundant, making up 19% of the total. *Anaeromyxobacter*, accounting for 9% of the bacteria, was the third most abundant genus. Among them, *Anaeromyxobacter* was found to be significantly more abundant in groups C101 and C104 compared to the other groups (*p* < 0.05).

#### 3.3.4. Structural Changes in the Microbial Communities in Rice Rhizosphere Soil

**Figure 2:**
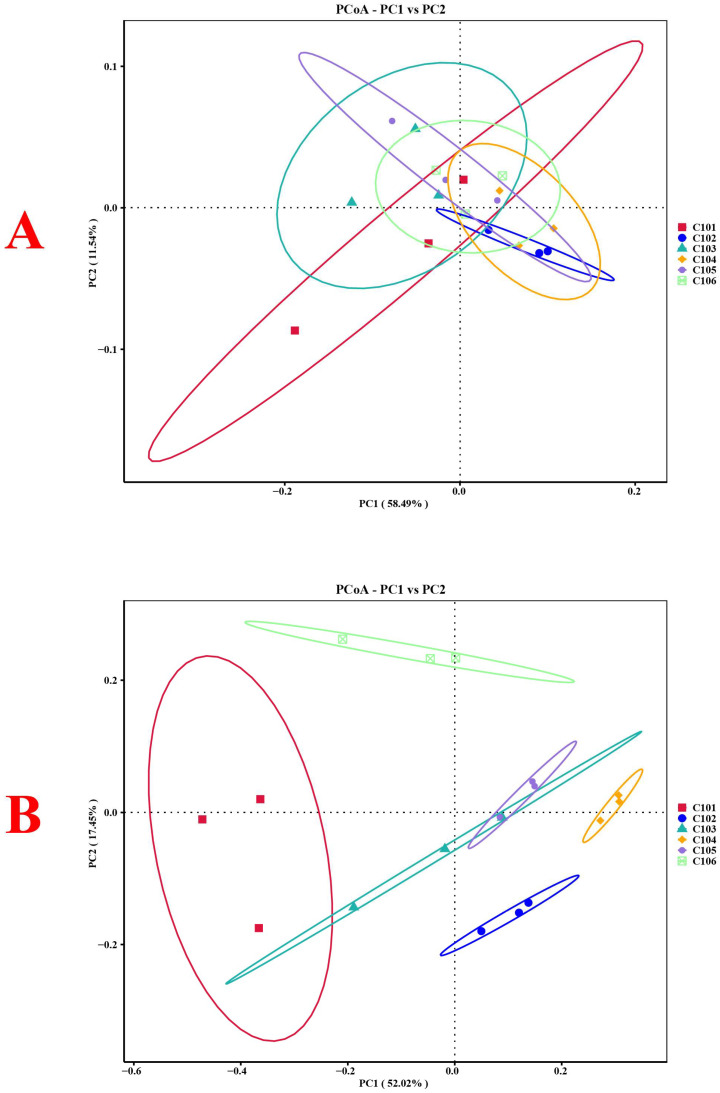
Analysis of β diversity of rhizosphere soil bacterial (**A**) and fungal (**B**) communities in different treatment groups based on PCoA.

**Figure 3:**
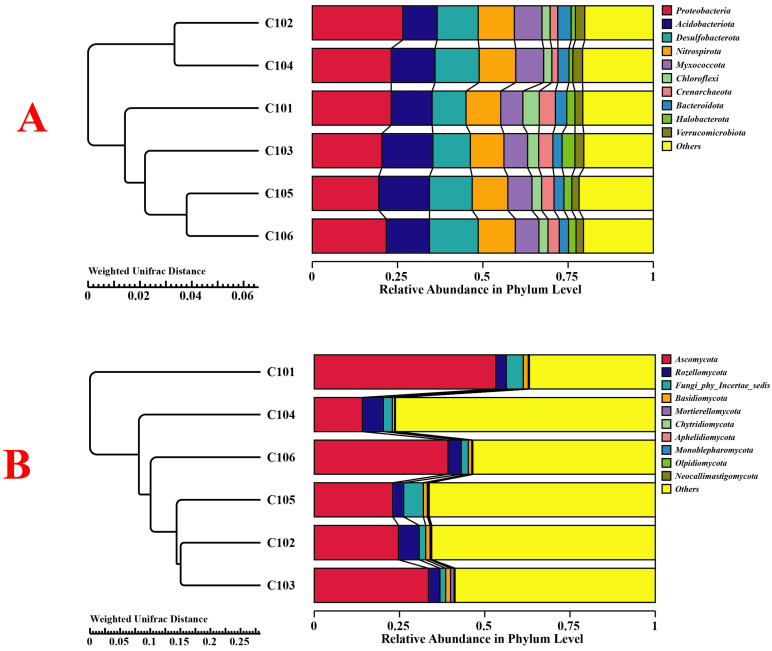
Changes in bacterial (**A**) and fungal (**B**) communities in different treatment groups based on NMDS analysis and weighted UniFrac distances.

The structural changes in bacterial and fungal communities in the different treatment groups were compared using principal coordinate analysis (PCoA) (Figure 2). The PC1 axis accounted for 58.49% of the differences in bacteria and 52.02% in fungi in the rhizosphere soil of rice, while the PC2 axis explained 11.54% and 17.45%, respectively. Overall, they explained 70.03% and 69.47% of the differences. The results indicate that rice with different NUE alters the community structure of its rhizosphere soil bacteria and fungi. From the perspective of the weighted UniFrac distance, the bacterial community structures of groups C105 and C106 were the most similar among all treatment groups, while the fungal community structures of groups C102 and C103 were the most similar (Figure 3). Similarly, the community structures of nitrogen-fixing bacteria in groups C105 and C106 were also the most similar (Figure S8).

#### 3.3.5. Functional Changes in Rice Rhizosphere Soil Microbes

The function of soil bacteria in the rice rhizosphere was predicted using PICRUSt2. According to the COG database, the function of COG0438 (glycosyltransferase involved in cell wall biosynthesis) showed a significant increase in group C103 when compared to groups C102 and C104 (*p* < 0.05). The function of COG0642 (signal transduction histidine kinase) was significantly increased in group C102 compared to groups C101 and C103 (*p* < 0.05). The function of COG0451 (nucleoside-diphosphate-sugar epimerase) was significantly reduced in group C101 compared to groups C103, C105, and C106 (*p* < 0.05). In the KO database, the function of K03088 (RNA polymerase sigma-70 factor) was significantly reduced in group C106 compared to group C103 (*p* < 0.05). The functions of K01990 (ABC-2 type transport system ATP-binding protein) and K01992 (ABC-2 type transport system permease protein) showed a significant increase in groups C103 and C105 in comparison to group C102 (*p* < 0.05). According to the pathway database, the function of P42-PWY (incomplete reductive TCA cycle) was significantly greater in groups C103 and C106 than in groups C101 and C104 (*p* < 0.05). The function of PWY-5104 (L-isoleucine biosynthesis IV) was significantly greater in groups C103, C105, and C106 than in groups C102 and C104 (*p* < 0.05). The function of BRANCHED-CHAIN-AA-SYN-PWY (the superpathway of branched amino acid biosynthesis) was significantly higher in groups C103 and C105 compared to groups C102 and C104 (*p* < 0.05).

FunGuild was used to analyze the metabolic pathways of fungal communities in rice rhizosphere soil. In the functional abundance results for Guild, Unassigned accounted for the highest proportion (78%), followed by Undefined_Saprotroph (18.39%) and Dung_Saprotroph-Undefined_Saprotroph (3.12%). Except for group C101, the abundance of Unassigned genes in group C102 was significantly higher compared to the other groups (*p* < 0.05). The abundance of Undefined_Saprotrophs in group C105 was significantly greater than in the other groups (*p* < 0.05). The abundance of Dung_Saprotroph-Undefined_Saprotroph in group C101 was significantly greater than in the other groups (*p* < 0.05). Among the functional abundance results for Mode, Unassigned and Saprotroph had the highest abundances, accounting for 74% and 26%, respectively. In group C104, the abundance of Unassigned bacteria was significantly greater than that in groups C101, C103, and C106 (*p* < 0.05). Except for group C106, the abundance of Saprotrophs in group C101 was significantly greater than that in the other groups (*p* < 0.05).

#### 3.3.6. Correlation Analysis of Environmental Factors and Microbial Communities in Rice Rhizosphere Soil

**Figure 4:**
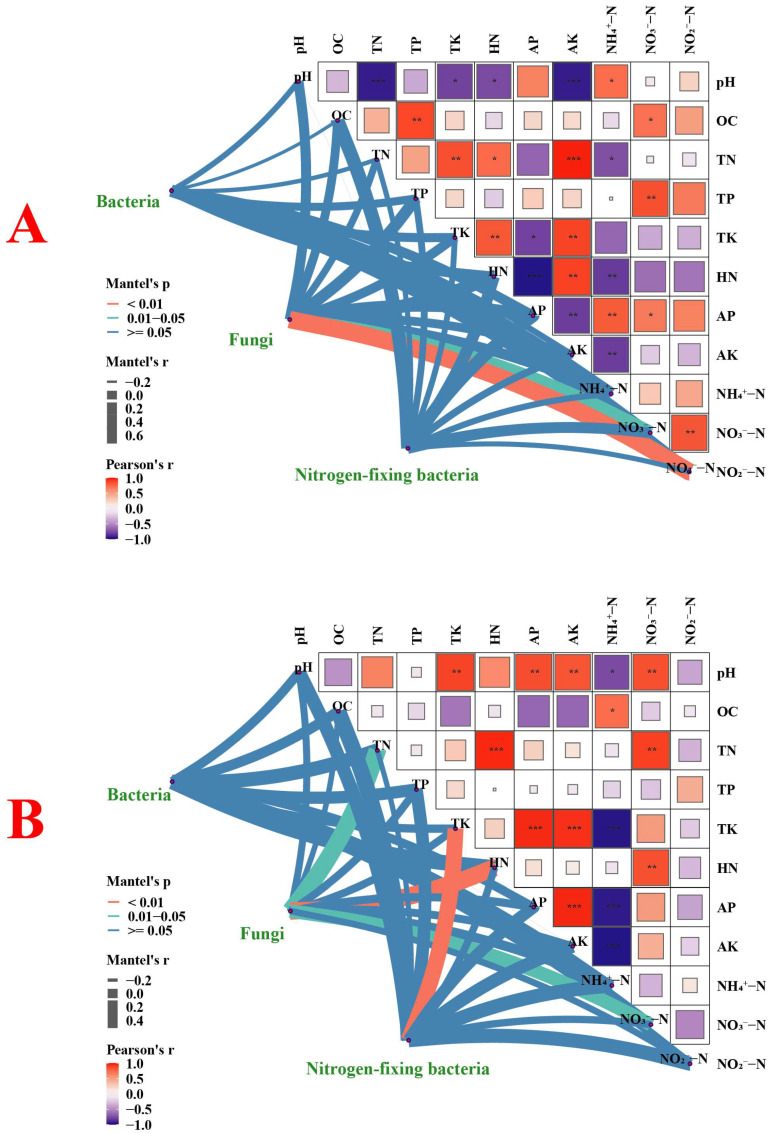
Mantle test and Pearson test of the relationship between soil rhizosphere bacterial, fungal, and nitrogen-fixing bacterial diversity and soil physical and chemical properties in high-NUE (**A**) and low-NUE (**B**) rice. Light blue lines indicate no significance (*p* > 0.05), light green lines indicate significance (*p* < 0.05), and orange lines indicate extreme significance (*p* < 0.01). Line thickness represents correlation strength. Red-purple rectangles denote correlation strength. A single asterisk denotes *p* < 0.05, a double asterisk denotes *p* < 0.01, and a triple asterisk denotes *p* < 0.001.

Figure 4 shows the correlation between microbial diversity and soil physical and chemical properties in rice with high and low NUE. As shown in Figure 4A, in high-NUE rice, the soil TN content is positively correlated with the AK content, while the soil HN content is negatively correlated with the AP content, and both correlations are highly significant (*p* < 0.001). In addition, there was no significant correlation between the diversity of high-NUE rice rhizosphere soil bacteria and nitrogen-fixing bacteria and the physical and chemical properties of the soil, while the diversity of fungi was significantly correlated with the contents of NO_3_^−^-N and NO_2_^−^-N in the soil (*p* < 0.05). As shown in Figure 4B, in low-NUE rice, there is a significant positive correlation between soil TN content and HN content, as well as between soil TK content and AK and AP content (*p* < 0.001). Furthermore, there is a significant positive correlation between soil AP content and AK content (*p* < 0.001). The soil NO_3_^−^-N content is significantly negatively correlated with the TK, AP, and AK content (*p* < 0.001). Additionally, there is a significant correlation between the fungal diversity in the rhizosphere soil of low-NUE rice and the soil HN content, as well as between the nitrogen-fixing bacterial diversity and the soil TK content (*p* < 0.01).

**Figure 5:**
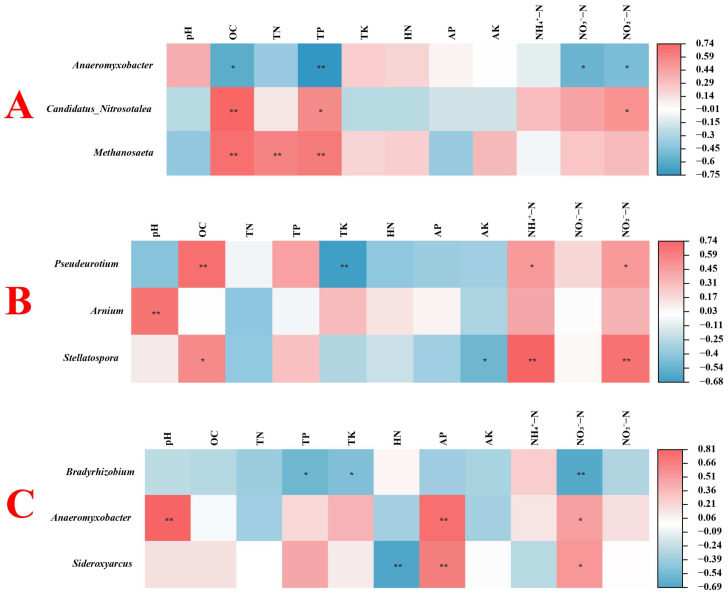
Analysis of the correlation between soil physical and chemical properties and rhizosphere bacteria (**A**), fungi (**B**), and nitrogen-fixing bacteria (**C**) in rice soil. One asterisk indicates *p* < 0.05, two asterisks indicate *p* < 0.01.

**Figure 6:**
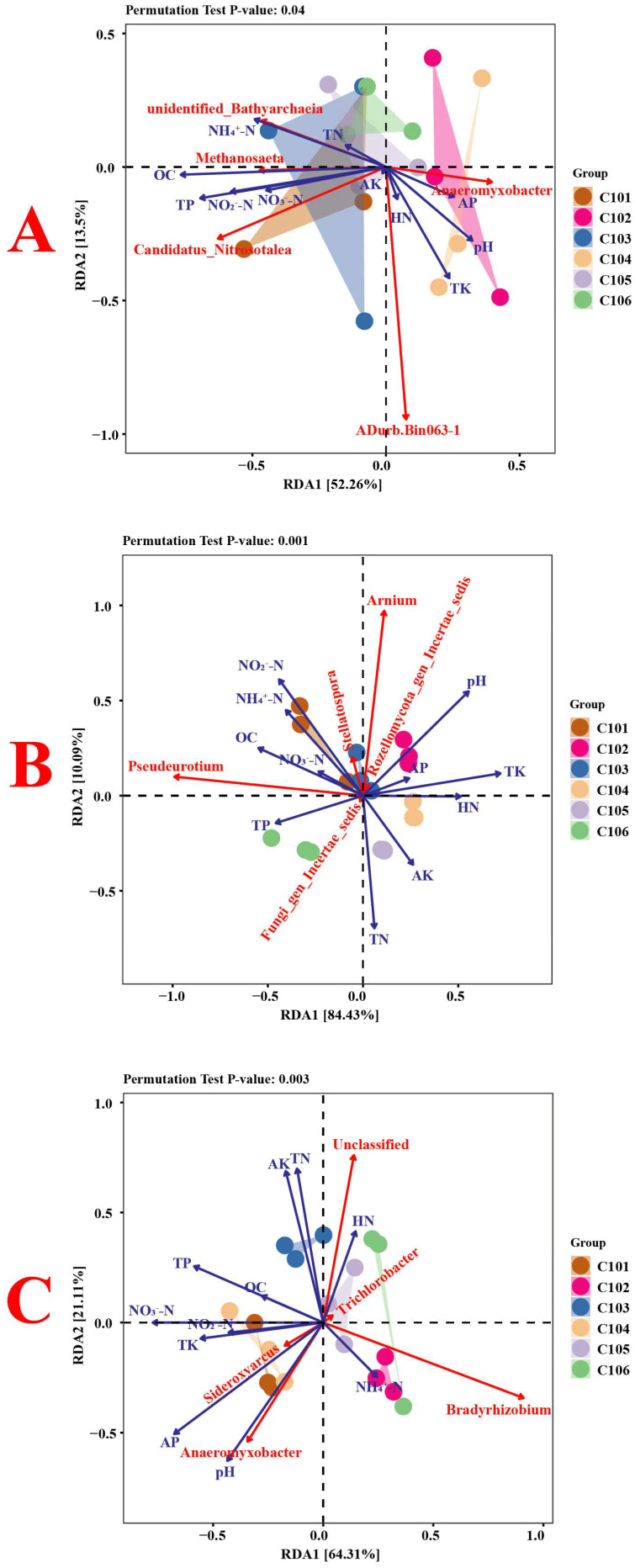
Redundancy analysis between soil physical and chemical properties and bacteria (**A**), fungi (**B**), and nitrogen-fixing bacteria (**C**) in the rhizosphere soil of rice.

To examine the relationship between environmental factors and rice rhizosphere soil microbes across all samples, the top five genera of bacteria, fungi, and nitrogen-fixing bacteria, as well as the soil’s physicochemical properties, were selected for correlation analysis (Figure 5) and redundancy analysis (Figure 6). In Figure 6A, RDA1 and RDA2 explained 52.26% and 13.50%, respectively, totaling 65.76%. *Methanosaeta* showed a significant positive correlation with soil OC content, TN content, and TP content (*p* < 0.05). Additionally, *Candidatus Nitrosotalea* showed a significant positive correlation with soil OC content, TP content, and NO_2_^−^-N content (*p* < 0.05). *Anaeromyxobacter* showed a significant negative correlation with soil OC content, TP content, NO_3_^−^-N content, and NO_2_^−^-N content (*p* < 0.05). In Figure 6B, RDA1 and RDA2 explained 84.43% and 10.09%, respectively, resulting in a total explanation rate of 94.52%. *Pseudeurotium* showed a significant positive correlation with soil OC content, NH_4_^+^-N content, and NO_2_^−^-N content, but a significant negative correlation with soil TK content (*p* < 0.05). Stellatospora showed a significant positive correlation with soil OC content, NH_4_^+^-N content, and NO_2_^−^-N content, but a significant negative correlation with soil AK content (*p* < 0.05). In addition, *Arnium* showed a strong positive correlation with soil pH (*p* < 0.05). In Figure 6C, RDA1 and RDA2 explained 64.31% and 21.11%, respectively, totaling 85.42%. Among them, *Sideroxyarcus* showed a significant positive correlation with soil AP content and NO_3_^−^-N content, and a significant negative correlation with the soil HN content (*p* < 0.05). *Anaeromyxobacter* was significantly positively correlated with the soil pH, AP content, and NO_3_^−^-N content (*p* < 0.05). *Bradyrhizobium* was significantly negatively correlated with the soil TP content, TK content, and NO_3_^−^-N content (*p* < 0.05).

## 4. Discussion

This study demonstrates that rice varieties with differing NUE reshape the composition and activity of the rhizosphere microbial community, eliciting distinct responses among bacteria, fungi, and nitrogen-fixing bacteria, which in turn may trigger cascading effects on nitrogen cycle dynamics in the soil. Contrary to the findings of Luo et al. [43], analysis of α-diversity in the rhizosphere soil microbial communities of rice revealed no significant changes in overall α-diversity, but this supports the hypothesis of functional redundancy in soil ecosystems [44]. Importantly, high-NUE rice fostered a unique microbial assemblage compared to low-NUE genotypes, despite minimal differences in bacteria and nitrogen-fixing bacteria α-diversity. Notably, the average abundance of the fungus *Arnium* in the rhizosphere soil microbiota of high-NUE rice was significantly higher than that in low-NUE rice, with an increase of 222.2% compared to low-NUE rice (*p* < 0.05). This suggests that *Arnium* may be a key fungal population mediating plant-microbe nitrogen collaboration. The dramatic enrichment of the saprotrophic fungus *Arnium* in the high-NUE rhizosphere suggests a potential mechanistic pathway for enhanced nitrogen availability. As a decomposer, *Arnium* likely facilitates the mineralization of organic nitrogen into plant-available ammonium, providing a direct and efficient nitrogen source for plant uptake. This transformation may be particularly crucial in the rhizosphere, where competition for nitrogen is intense. High-NUE cultivars selectively recruit *Arnium* through root exudates, effectively ‘outsourcing’ the task of organic matter decomposition to gain a competitive advantage in nitrogen acquisition. Furthermore, as a fungal hub, *Arnium* may interact synergistically with other nitrogen-cycling bacteria, structuring a microbial community that collectively optimizes nitrogen cycling processes to benefit the host plant. Additionally, the key microbial populations (*Anaeromyxobacter*, *Arnium*, and *Bradyrhizobium*) exhibited by the C102 group in high-NUE rice may be associated with nitrogen cycling and plant growth promotion [45,46,47]. The analysis of microbial β diversity revealed significant changes in the community structure of rhizosphere soil microorganisms in rice, affecting fungal communities more than bacterial communities, similar to Bai et al.’s findings [48]. This suggests that NUE-driven microbial reorganization is achieved through changes in microbial structure and function rather than alterations in diversity itself.

Soil physicochemical properties are important mediators of the relationship between NUE and microbial dynamics [49]. Among nitrogen-fixing bacteria, *Anaeromyxobacter* was positively correlated with soil pH, AP, and NO_3_^−^-N content, which is consistent with its known role in nitrate reduction [50]. In contrast, the negative correlations between *Bradyrhizobium* and TP, TK, and NO_3_^−^-N may reflect competition with other nitrogen-cycling microbes under nutrient-rich conditions [51]. Importantly, soil TN and HN contents were the main differentiating factors between high-NUE and low-NUE rice. High-NUE rice maintained lower TN and higher HN, indicating high nitrogen uptake efficiency and effective conversion to available plant forms. This process may be mediated by rhizosphere microbes. This was further confirmed by a significant negative correlation between HN and AP in high-NUE rice, indicating that microbial activities drove more stringent nutrient coregulation. Low-NUE rice showed a notable positive correlation between soil TN and HN contents, which may suggest low nitrogen fixation efficiency. Additionally, NH_4_^+^-N had a significant negative correlation with TK, AK, and AP. This relationship indicates that potassium and phosphorus may serve as synergistic regulators of nitrogen retention [52]. Moreover, changes in environmental factors also affect the diversity and richness of soil microbes [53,54]. Redundancy analysis revealed that the response of key microbial genera to nitrogen form differed among rice plants with different NUE, indicating that the nitrogen form, not just the total nitrogen content, shapes microbial niches. The soil NO_3_^−^-N and NO_2_^−^-N contents were significantly correlated with the diversity of the fungal community in high-NUE rice. In low-NUE rice, there was a significant correlation between the diversity of the fungal community and the soil TN, HN, and NO_3_^−^-N content (*p* < 0.05). This phenomenon revealed the ecological strategy differentiation of the rice rhizosphere fungal community in response to nitrogen forms, driven by differences in NUE, indicating that different forms of nitrogen have different regulatory effects on microbial communities.

Functional prediction further elucidated the metabolic basis of the difference in rice NUE. The increase in functions such as the incomplete reductive TCA cycle (P42-PWY) and the superpathway of branched amino acid biosynthesis (BRANCHED-CHAIN-AA-SYN-PWY) in group C103 may be critical for plant stress resistance and growth. The upregulation of ABC transporter genes (K01990, K01992) in groups C103 and C105 indicates that they enhance the nutrient capture capacity, which may be a significant feature of microbial communities associated with efficient nutrient utilization in plants. For fungi, the enrichment of Undefined_Saprotrophs in low-NUE rice means that organic matter degradation is greater, which may compensate for the low-NUE of the host plants.

Traditional nitrogen fertilizer management strategies primarily rely on applying large quantities of chemical fertilizers to ensure crop needs are met, but this approach incurs significant environmental costs. Our research indicates that through crop genotype selection or microbial community management, rhizosphere microorganisms can be domesticated or guided to reorganize into an efficient natural bioreactor. This reactor optimizes nitrogen transformation, sequestration, and supply processes, thereby reducing nitrogen fertilizer application rates and environmental leaching. Microbial community reorganization extends beyond optimizing nitrogen cycling. Microbial communities associated with high-NUE rice may enhance organic matter decomposition and nutrient cycling capacity. This implies that cultivating high-NUE crop varieties could progressively improve soil physical, chemical, and biological properties by altering their rhizosphere microbiomes. This approach enhances long-term soil health and fertility, breaking the vicious cycle of soil degradation caused by excessive fertilization.

## 5. Conclusions

This study investigated the changes in soil physical and chemical properties, rhizosphere soil microbial communities, and their functions in rice varieties with different NUE levels. The results indicate that rice varieties with different NUE levels reshaped the structure and function of rhizosphere soil microbial communities, but the overall diversity of rhizosphere soil microbial communities did not undergo significant changes. Specifically, there were no significant changes in the α-diversity between rhizosphere soil bacteria and nitrogen-fixing bacteria among rice varieties with different NUE levels. However, in rhizosphere soil fungal communities, the α-diversity index was generally higher in rice varieties with high-NUE than in those with low-NUE. Notably, the average abundance of the fungus *Arnium* in the rhizosphere soil microorganisms of high-NUE rice was significantly higher than that in low-NUE rice, with an increase of 222.2% compared to low-NUE rice. Redundancy analysis and correlation analysis results indicate that in high-NUE rice, soil NO_2_^−^-N content is the most critical factor influencing fungal community diversity in the rhizosphere soil, while in low-NUE rice, soil HN and TK contents are the most critical factors influencing fungal and nitrogen-fixing bacterial community diversity, respectively. The results of this study will help us understand the effects of NUE differences in rice on soil and rhizosphere microbial communities. This study established the connection between NUE-driven rhizosphere microbial community restructuring and soil nitrogen dynamics, providing a theoretical foundation for precision nitrogen fertilizer management strategies based on rhizosphere microbial regulation.

## Figures and Tables

**Table 1:** Soil physicochemical properties of each rice treatment group.

	pH	OC (g/kg)	TN (g/kg)	TP (g/kg)	TK (g/kg)	HN (mg/kg)	AP (mg/kg)	AK (mg/kg)	NH4^+^-N (mg/kg)	NO_3_^−^-N (mg/kg)	NO_2_^−^-N (mg/kg)
C101	6.07 ± 0.05 a	31.43 ± 0.84 a	1.93 ± 0.02 e	0.81 ± 0.03 a	13.78 ± 0.3 c	233.49 ± 1.91 d	1.54 ± 0.04 b	85.68 ± 1.46 e	6.87 ± 0.54 a	4.90 ± 0.23 a	0.25 ± 0.02 a
C102	6.07 ± 0.03 a	29.40 ± 0.64 b	1.82 ± 0.03 f	0.63 ± 0.02 c	14.09 ± 0.2 c	262.93 ± 2.34 b	1.29 ± 0.07 cd	96.28 ± 1.97 d	6.42 ± 0.40 ab	1.78 ± 0.11 f	0.20 ± 0.01 b
C103	5.89 ± 0.04 c	31.17 ± 0.73 a	3.05 ± 0.04 a	0.79 ± 0.03 ab	14.79 ± 0.3 b	282.53 ± 5.90 a	1.18 ± 0.05 e	142.14 ± 1.02 a	5.60 ± 0.40 c	3.20 ± 0.17 d	0.22 ± 0.02 b
C104	6.06 ± 0.03 a	29.51 ± 0.79 b	2.82 ± 0.03 c	0.76 ± 0.03 b	15.31 ± 0.3 a	250.20 ± 4.08 c	1.64 ± 0.04 a	125.20 ± 1.98 b	4.38 ± 0.14 d	3.55 ± 0.15 c	0.19 ± 0.02 b
C105	5.98 ± 0.02 b	30.31 ± 0.34 ab	2.95 ± 0.03 b	0.75 ± 0.02 b	13.87 ± 0.3 c	265.37 ± 4.52 b	1.36 ± 0.04 c	113.83 ± 2.99 c	5.60 ± 0.35 c	3.98 ± 0.13 b	0.19 ± 0.01 b
C106	5.76 ± 0.03 d	30.34 ± 0.54 ab	2.71 ± 0.03 d	0.76 ± 0.03 ab	13.17 ± 0.3 d	240.44 ± 6.23 d	1.24 ± 0.03 de	110.64 ± 1.63 c	5.79 ± 0.18 bc	2.45 ± 0.07 e	0.20 ± 0.01 b

Data are means ± standard error (n = 3). Different lowercase letters indicate significant differences (*p* < 0.05) (n = 3).

**Table 2:** The NUE of each rice treatment group.

	Normal Fertilization Yield (kg)	Nitrogen-Free Treatment Yield (kg)	NUE (kg/kg)
C101	732.10 ± 18.30	412.08 ± 14.33	26.67 ± 1.72 a
C102	696.23 ± 16.61	374.62 ± 7.64	26.80 ± 0.99 a
C103	720.35 ± 7.15	403.19 ± 5.25	26.43 ± 0.50 a
C104	615.90 ± 6.34	475.26 ± 10.61	11.72 ± 1.39 c
C105	543.20 ± 20.55	364.15 ± 5.58	14.92 ± 1.25 b
C106	645.75 ± 23.81	507.96 ± 19.46	11.48 ± 2.37 c

Data are means ± standard error (n = 3). Different lowercase letters indicate significant differences (*p* < 0.05) (n = 3).

## Data Availability

The original contributions presented in this study are included in the article/Supplementary Material. Further inquiries can be directed to the corresponding author.

## Supplementary Materials

The following supporting information can be downloaded at: https://www.mdpi.com/article/10.3390/microorganisms13092215/s1, Figure S1: Phylogenetic relationships and abundance of the 100 most common bacterial genera in rice rhizosphere soil; Figure S2: Phylogenetic relationships and abundance of the 100 most common fungal genera in rice rhizosphere soil; Figure S3: Phylogenetic relationships and abundance of the 100 most common nitrogen-fixing bacterial genera in rice rhizosphere soil; Figure S4: Box plots of α diversity indices of rhizosphere soil nitrogen-fixing bacteria in different treatment groups; Figure S5: Relative abundance of the top 10 bacterial taxa in rice rhizosphere soil at the phylum (A), class (B), order (C), and genus (D) levels; Figure S6: Relative abundance of the top 10 fungal taxa in rice rhizosphere soil at the phylum (A), class (B), order (C), and genus (D) levels; Figure S7: Relative abundance of the top 10 nitrogen-fixing bacteria taxa in rice rhizosphere soil at the phylum (A), class (B), order (C), and genus (D) levels; Figure S8: Changes in nitrogen-fixing bacteria communities in different treatment groups based on NMDS analysis and weighted UniFrac distances; Method: Sequencing and analysis methods. Refs. [55,56,57,58] can be found in Supplementary Materials.
