# Supplementary material for: The Reorganization of Rice Rhizosphere Microbial Communities Driven by Nitrogen Utilization Efficiency and the Regulatory Mechanism of Soil Nitrogen Cycling
Source: Microorganisms. 2025 Sep 22;13(9):2215. doi: 10.3390/microorganisms13092215 (PMC12472575; doi:10.3390/microorganisms13092215)
Supplement: Supplementary file 1 [file microorganisms-13-02215-s001.zip › Supplementary Figures.pdf]

Supplementary Materials

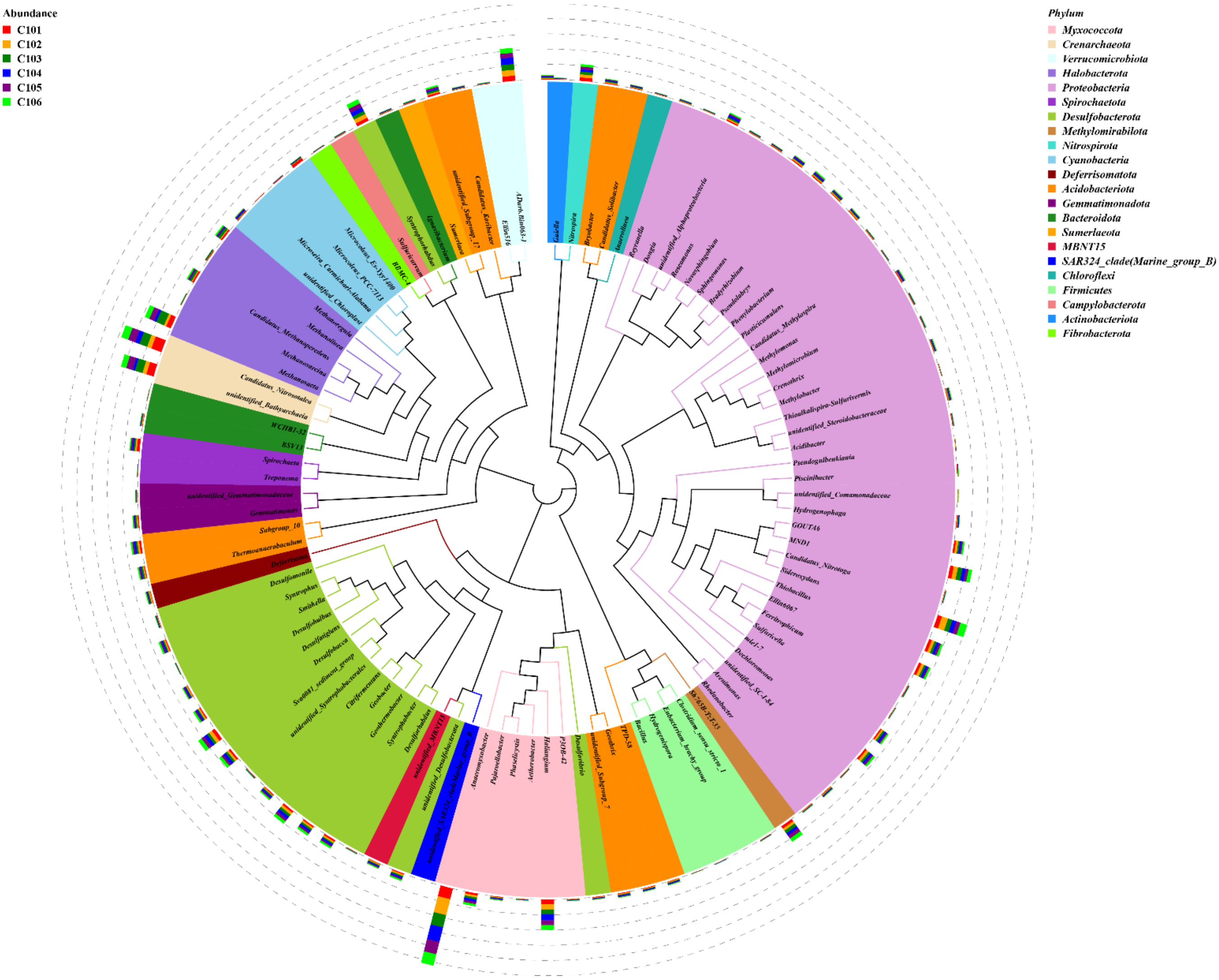

Figure S1. Phylogenetic relationships and abundance of the 100 most common bacterial genera in rice rhizosphere soil.

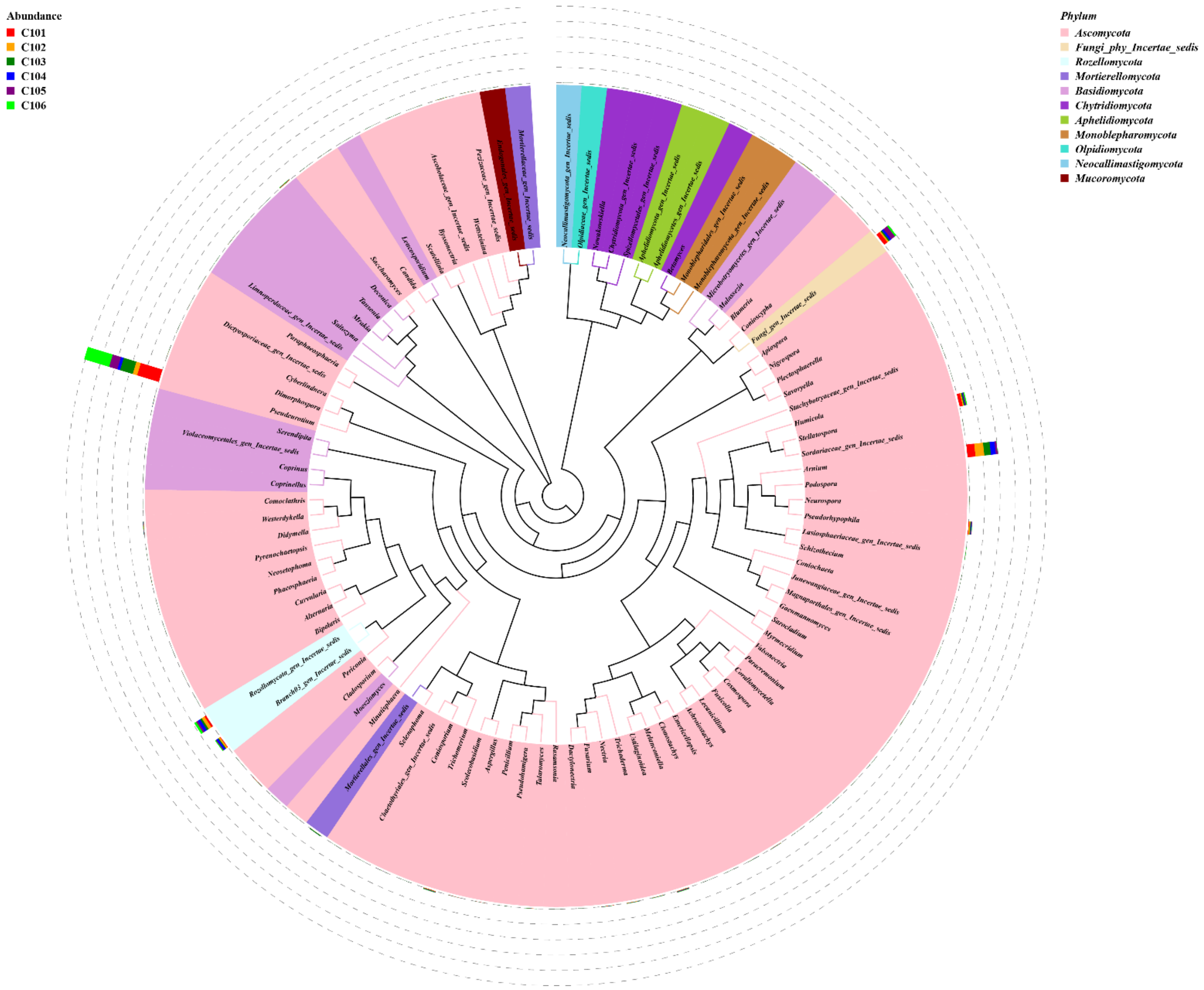

Figure S2. Phylogenetic relationships and abundance of the 100 most common fungal genera in rice rhizosphere soil.

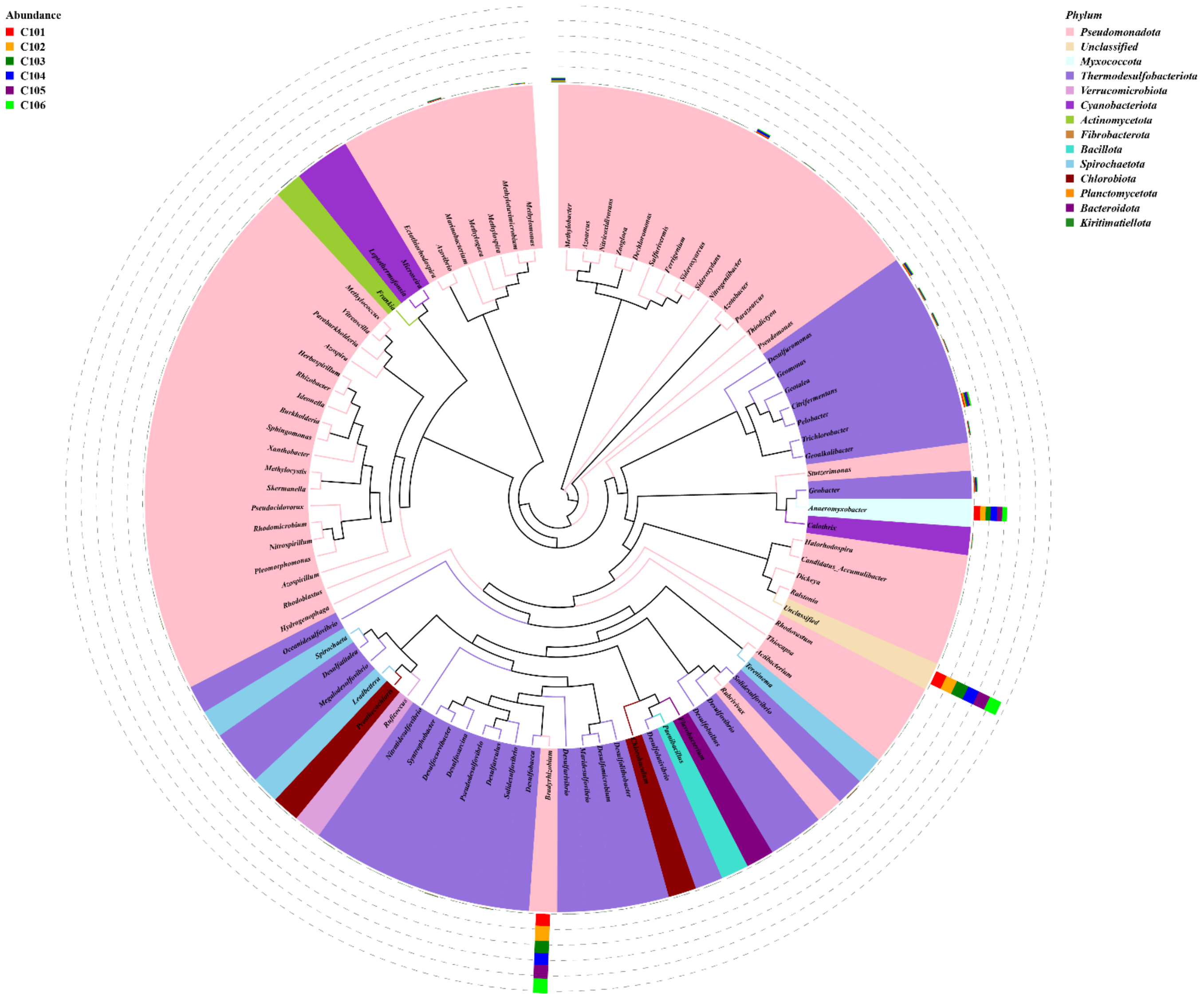

Figure S3. Phylogenetic relationships and abundance of the 100 most common nitrogen-fixing bacterial genera in rice rhizosphere soil.

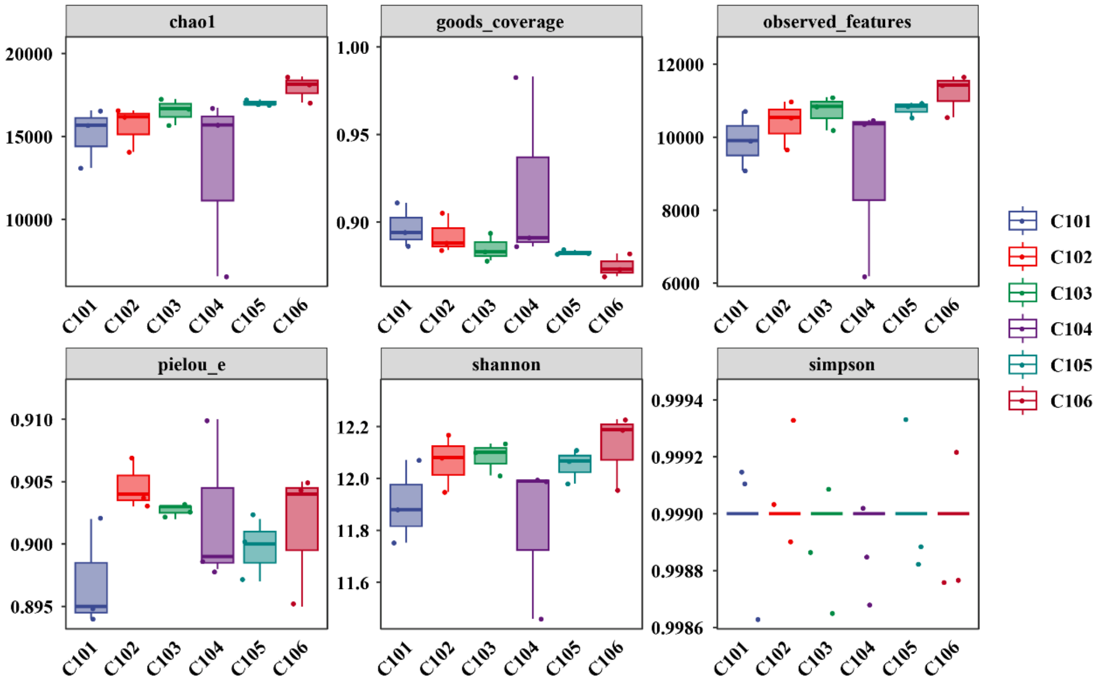

Figure S4. Box plots of α diversity indices of rhizosphere soil nitrogen-fixing bacteria in different treatment groups.

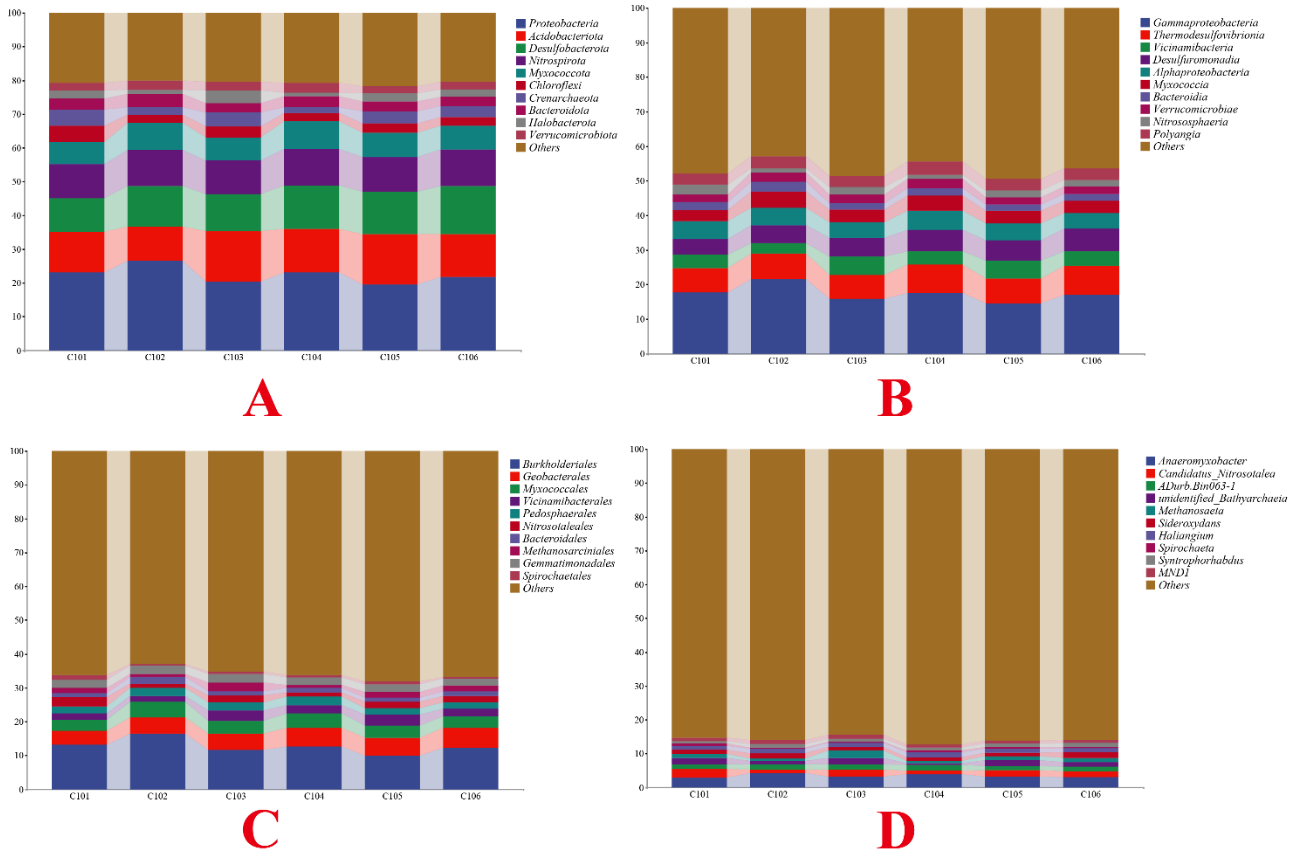

Figure S5. Relative abundance of the top 10 bacterial taxa in rice rhizosphere soil at the phylum (A), class (B), order (C), and genus (D) levels.

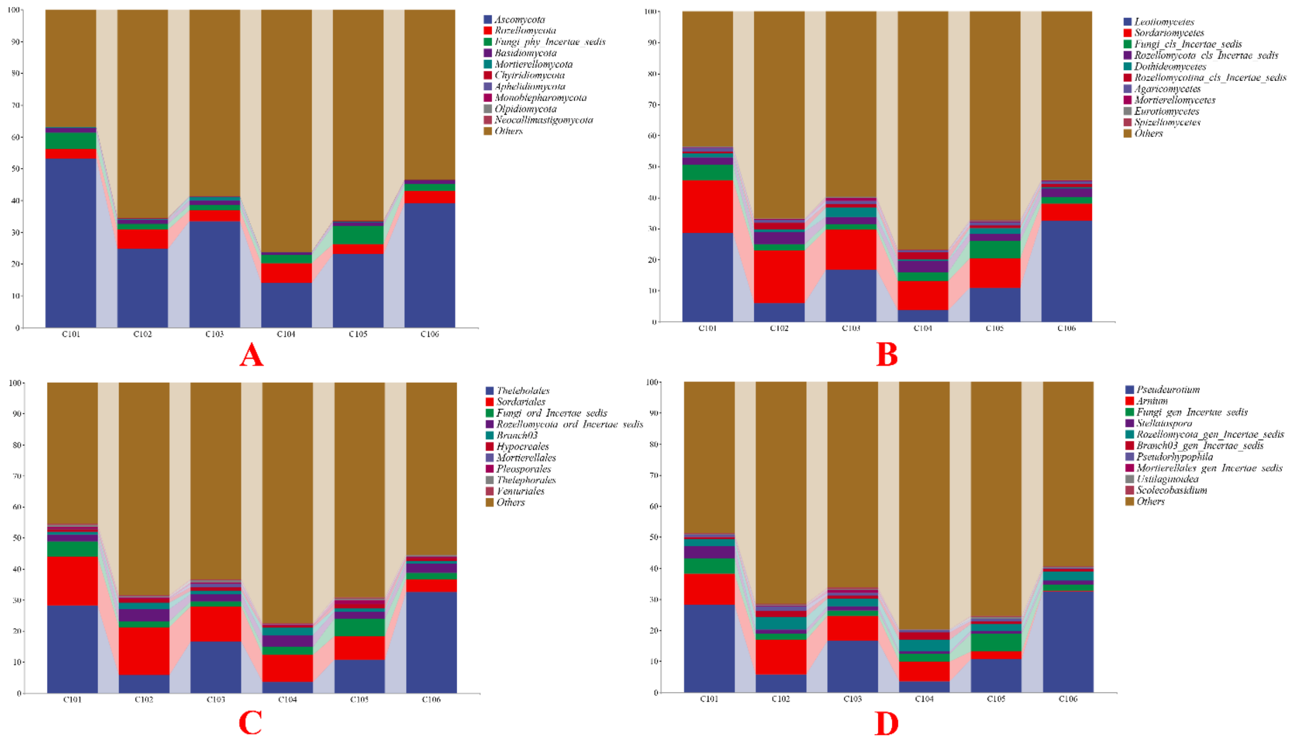

Figure S6. Relative abundance of the top 10 fungal taxa in rice rhizosphere soil at the phylum (A), class (B), order (C), and genus (D) levels.

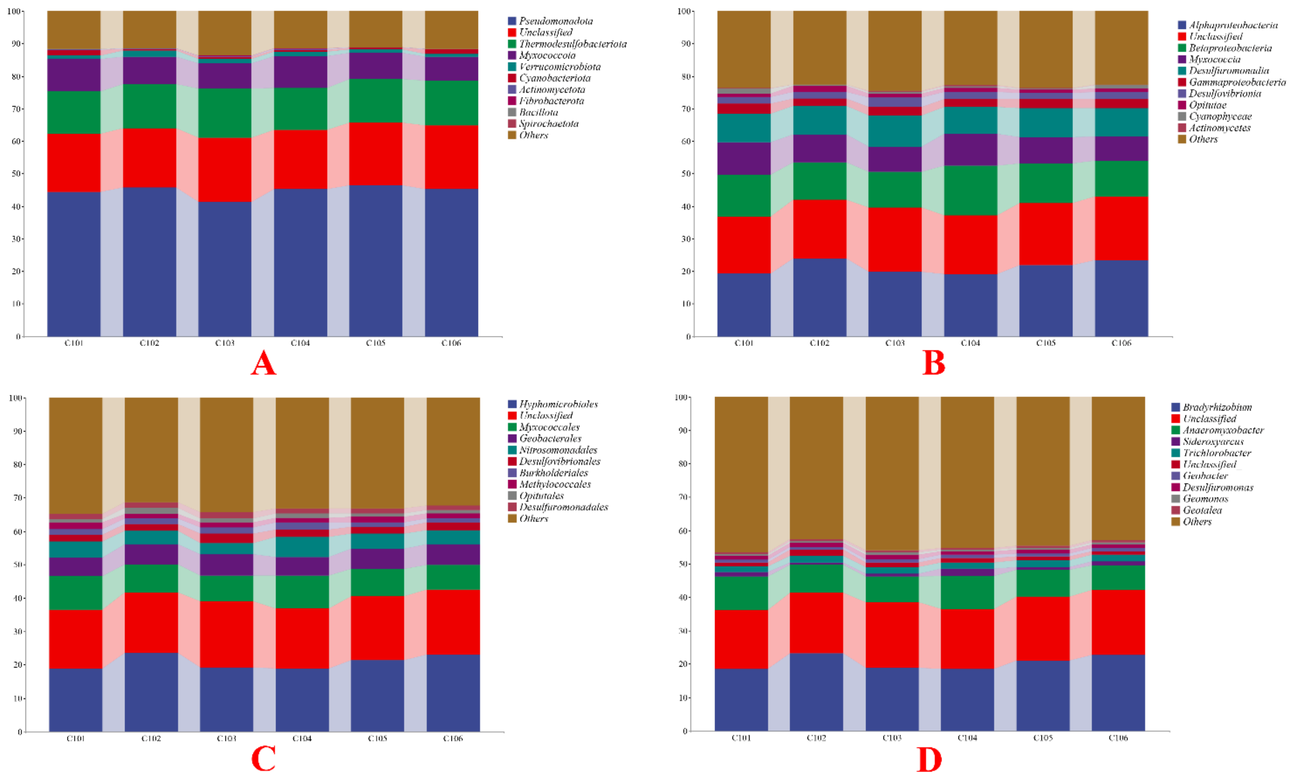

Figure S7. Relative abundance of the top 10 nitrogen-fixing bacteria taxa in rice rhizosphere soil at the phylum (A), class (B), order (C), and genus (D) levels.

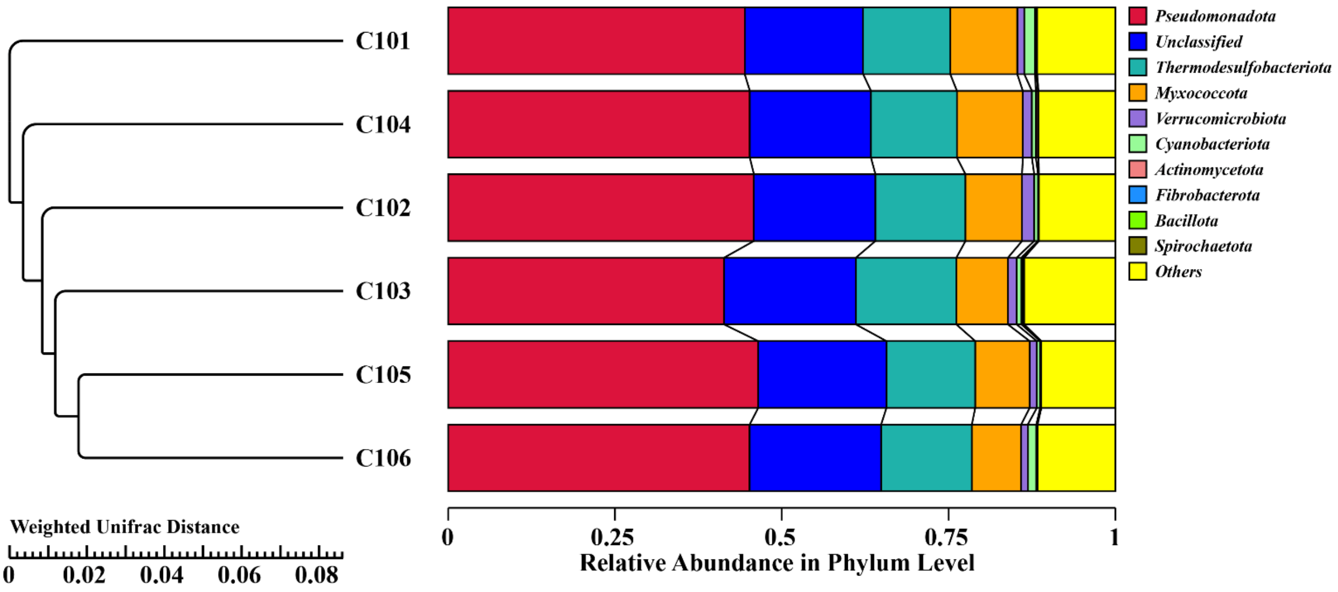

Figure S8. Changes in nitrogen-fixing bacteria communities in different treatment groups based on NMDS analysis and weighted UniFrac distances.
