# Supplementary material for: The Reorganization of Rice Rhizosphere Microbial Communities Driven by Nitrogen Utilization Efficiency and the Regulatory Mechanism of Soil Nitrogen Cycling
Source: Microorganisms. 2025 Sep 22;13(9):2215. doi: 10.3390/microorganisms13092215 (PMC12472575; doi:10.3390/microorganisms13092215)
Supplement: Supplementary file 1 [file microorganisms-13-02215-s001.zip › Supplementary Method.pdf]

Sequencing and analysis methods

Sequencing libraries were generated and indexes were added. The library was checked with Qubit and real-time PCR for quantification and bioanalyzer for size distribution detection. Quantified libraries were pooled and sequenced on Illumina platforms, according to effective library concentration and data amount required. Paired- end reads were assigned to samples based on their unique barcode and truncated by cutting off the barcode and primer sequence. Paired-end reads were merged using FLASH (V1.2.11, http://ccb.jhu.edu/software/FLASH/) [55], a very fast and accurate analysistool, which was designed to merge paired end reads when at least some of the reads overlap theread generated from the opposite end of the same DNA fragment, and the splicing sequences were called raw tags. Quality filtering on the raw tags were performed using the fastp (Version 0.23.1) software to obtain high-quality Clean Tags [56]. The tags were compared with the reference database (Silva database (16S), https://www.arb-silva.de/; Unite Database (ITS), https://unite.ut.ee/) to detect chimera sequences, And the effective tags were obtained by removing the chimera sequences with the vsearch package (V2.16.0, https://github.com/torognes/vsearch) [57]. For the Effective Tags obtained previously, denoise was performed with DADA2 or deblur module in the QIIME2 software to obtain initial ASVs (Amplicon Sequence Variants) [58]. Species annotation was performed using QIIME2 software. For 16S, the annotation database is Silva Database, while for ITS, it is Unite Database. In order to study phylogenetic relationship of each ASV and the differences of the dominant species among different groups, multiple sequence alignment was performed using QIIME2 software. The absolute abundance of ASVs was normalized using a standard of sequence number corresponding to the sample with the least sequences. Subsequent analysis of alpha diversity and beta diversity were all performed based on the output normalized data. Top 10 taxa of each samples at each taxonomic ranks were selected to plot the distribution histogram of relative abundance in Perl through SVG function. One hundred genera with the highest abundance in the samples were selected and performed sequence alignment to draw the phylogenetic tree in perl with SVG function. Species accumulation boxplot can be used to visualize, which performed with vegan package

in R software. PCoA analysis was displayed by ade4 package and ggplot2 package in R software (Version 4.0.3). NMDS analysis was implemented through R software with ade4 package and ggplot2 package. PICRUSt (V1.1.4) is a package in R and is mainly used to predict the metagenomic functions based on marker genes. PICRUSt2 (V2.3.0) is the improved version of PICRUSt. Tax4Fun (V0.3.1) is a R package that is widely used for intestinal and soil samples. In general, it can supply more accurate results when compared with PICRUSt, especially for soil samples. BugBase is an excellent tool to discover the phenyotpe of microogranisms. It can classify the microbial communities according to seven phenotypes: Gram Positive, Gram Negative, Biofilm Forming, Pathogenic, Mobile Element Containing, Oxygen Utilization, including Aerobic, Anaerobic, and Cultivable Anaerobic, and Oxidative Stress Tolerance. FunGuild is an exce lent tool through python when working with fungi samples. FAPROTAX through python can play a great role when elucidating the possible biochemical processes and elements in play. To investigate symbiotic relationships among species and reveal the impact of environmental factors on community structure, Spearman's correlation analysis and Redundancy Analysis (RDA) were employed to assess the association between environmental factors and species abundance. All graphical representations and analyses were conducted using R software.

References

1. Magoč, T.; Salzberg, S.L. FLASH: fast length adjustment of short reads to improve genome assemblies. *Bioinformatics* **2011**, *27*, 2957–2963.
2. Bokulich, N.A.; Subramanian, S.; Faith, J.J.; Gevers, D.; Gordon, J.I.; Knight, R.; Mills, D.A.; Caporaso, J.G. Quality-filtering vastly improves diversity estimates from Illumina amplicon sequencing. *Nat. Methods* **2013**, *10*, 57–59.
3. Edgar, R.C.; Haas, B.J.; Clemente, J.C.; Quince, C.; Knight, R. UCHIME improves sensitivity and speed of chimera detection. *Bioinformatics* **2011**, *27*, 2194–2200.
4. Wang, Y.; Guo, H.; Gao, X.; Wang, J. The Intratumor Microbiota Signatures Associate With Subtype, Tumor Stage, and Survival Status of Esophageal Carcinoma. *Front. Oncol.* **2021**, *11*, 754788.
